# Dataset on sub-daily vertical profiles of physicochemical parameters and chlorophyll concentration in El Val reservoir, together with its daily meteorological data, storage state and downstream flow (2018–2022)

**DOI:** 10.1016/j.dib.2024.110839

**Published:** 2024-08-14

**Authors:** María Castrillo, Fernando Aguilar, Daniel García-Díaz

**Affiliations:** Institute of Physics of Cantabria (IFCA-(CSIC-UC)), Avda. Los Castros s/n, 39005 Santander, Cantabria, Spain

**Keywords:** Monitoring, Eutrophication, Thermal stratification, Water quality

## Abstract

The dataset addressed in this article contains parameters about El Val reservoir (province of Zaragoza, Spain). It includes physicochemical variables, the water level, the stored water volume, its meteorological conditions and the flow rate of its effluent, the Queiles River, a few metres downstream of the dam. The El Val reservoir stores water from the Val River, but it also receives water from the Queiles River through a pipeline and from several ravines. The dam releases on the Queiles River, which is a tributary of the Ebro River (the second one in Spain in length and discharge rate). A multiparametric probe (aquaDam, Adasa Systems), hanging from a structure located in the dam, every 6 h makes a vertical profile taking the measurements at each metre of depth from the surface to approximately 573 m above sea level (m.a.s.l.), in other words, between 2 and 3 m above the bottom outlet. This probe collects data of water temperature, pH, ORP, conductivity, dissolved oxygen, turbidity and chlorophyll concentration. Meteorological data are collected in the nearest weather station, located in the municipality of Los Fayos which is about 500 m downstream of the dam. These include daily accumulated precipitation, daily maximum and average solar irradiance, daily maximum, minimum and average air temperature and daily average wind speed. The water level and volume of stored water and the flow rate of the Queiles River are collected in the El Val monitoring station and the Queiles River gauge station respectively, and are also provided on a daily basis.

These data are useful to feed deterministic, data driven or hybrid hydrological models with different purposes, like the identification of the impact of meteorological conditions on the physicochemical properties of the reservoir as well as the assessment of different management strategies in the reservoir.

This is a data article that additionally supports the work published in Ecological Informatics [1] where the use of common and readily available open data is promoted through its use to feed data driven models, in particular to infer the depth of the thermocline in reservoirs that are periodically or permanently thermally stratified. In that article a dataset derived from the one presented in this article is used.

Specifications Table

**Table:** 

Subject	Hydrology and Water quality
Specific subject area	Time series of sub-daily physicochemical parameters and chlorophyll concentration in El Val reservoir, together with its daily meteorological data, storage state and downstream flow.
Type of data	Table, filtered, curated
Data collection	The original data are continuously collected by the Confederación Hidrográfica del Ebro (CHE) and published in real time. Then, the CHE curates the data that are finally available to the citizens under request by selecting specific dates and variables. In order to facilitate their availability and reuse, these data from 2018 to 2022 have been gathered, pre-processed and packaged in the form of datasets. Specifically they were collected from the Ebro Automatic Water Quality Information System (SAICA Ebro by its initials in Spanish) and the Ebro Automatic Hydrographic Information System (SAIH Ebro by its initials in Spanish), as it will be further explained in the experimental design, materials and methods.
Data source location	El Val reservoir Town, region: Los Fayos, Zaragoza Country: Spain Latitude and longitude for collected samples/data: 41.876, –1.787
Data accessibility	Repository name: Environmental Data Initiative (EDI) Data identification number: https://doi.org/10.6073/pasta/00be57c4f397606fb4897cd257b640cc
Related research article	María Castrillo, Fernando Aguilar, Daniel García-Díaz. 2024. A data-driven approach for the assessment of the thermal stratification of reservoirs based on readily available data. Ecological Informatics, 102,672, https://doi.org/10.1016/j.ecoinf.2024.102672.

## 1 Value of the Data

• The dataset includes five complete years of frequently measured data that includes different parameters related to water quality in a freshwater reservoir. The data are provided as four daily vertical profiles. Additionally, it includes external related parameters like meteorological ones, reservoir water level and volume, and downstream flow rate.
• This data can be used by researchers, students, and lecturers to conduct their research on hydrology and water quality. The diversity of parameters and its frequency is helpful for performing hydrodynamics and water quality modelling. In particular, the vertical profiling can support a better understanding of the reservoir dynamics and potential algal blooms.
• This data can serve as an example for water management authorities on how to put at the disposal of citizens, and researchers in particular, the information that they collect and its benefits for the development of open science.
• This dataset can be used for analysis using statistical methods or as training input data for machine learning techniques, such as correlation analysis, neural networks, clustering, time-series related models [1].

## 2 Background

Water bodies are increasingly suffering from diverse pressures like nutrient pollution leading to eutrophication and consequently to the excessive growth of algae. Monitoring plays a key role in the contribution to water sustainability [2]. In 2022 the United Nations’ (UN) [3] recognized that the lack of monitoring is limiting the identification of water quality issues and the implementation of proactive mitigation measures. Therefore, monitoring together with the availability of the data is crucial to go further in the protection of water. In particular, El Val reservoir is part of the Registry of Protected Areas according to the Water Framework Directive (2000/60/EC): Natura 2000 Network ES0000297, but it suffers eutrophication episodes.

According to the World Health Organization (WHO) [4], various parameters related to water quality intended for different types of human activity, particularly for consumption, have been identified. They can serve as indicators to determine if the water is suitable for consumption and additionally they can be used to infer other parameters of phenomena. The availability of Open Data fosters the development of new ways of using them, thus accelerating research and stimulating innovation and knowledge transfer.

## 3 Data Description

**Fig. 1:**
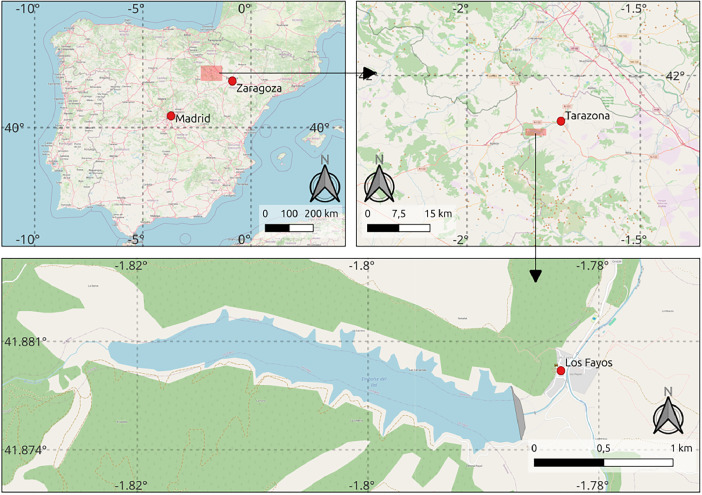
Map of the study site [1]. Source: OpenStreetMap.

The dataset addressed in this article contains the physicochemical parameters and chlorophyll concentration of El Val reservoir (province of Zaragoza, Spain), together with its meteorological conditions, the water level, the stored volume and the flow rate of its effluent, the Queiles River, a few metres downstream of the dam. The El Val reservoir stores water from the Val River, but it also receives water from the Queiles River through a pipeline and from several ravines. The dam releases on the Queiles River, which is a tributary of the Ebro River (the second one in Spain in length and discharge rate). A map of the reservoir area and its location in Spain is shown in Fig. 1.

**Fig. 2:**
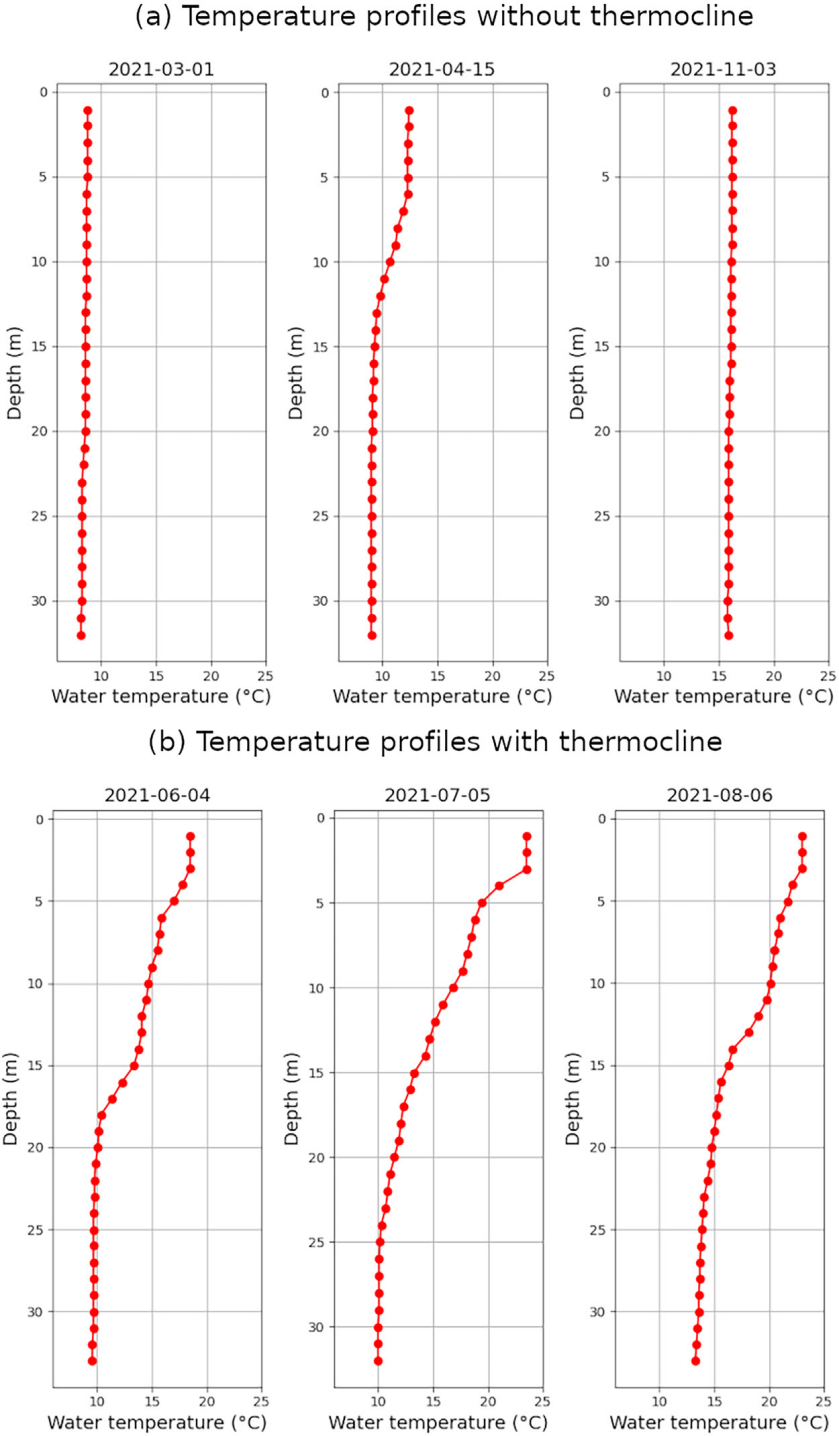
Vertical temperature profiles without (a) and with thermocline (b).

In order to facilitate their reuse, the collected data have been restructured and organised in different files according to its theme and sampling frequency. The first file includes the data related to the sub-daily vertical profiles of water quality parameters; the second one groups the set of daily meteorological variables; the third one lists the daily level and volume of the reservoir and finally the fourth one represents the water flow of the Queiles river downstream of the dam. An example of vertical profiles can be found in Fig. 2, both without (a) or with thermocline (b).

**Fig. 3:**
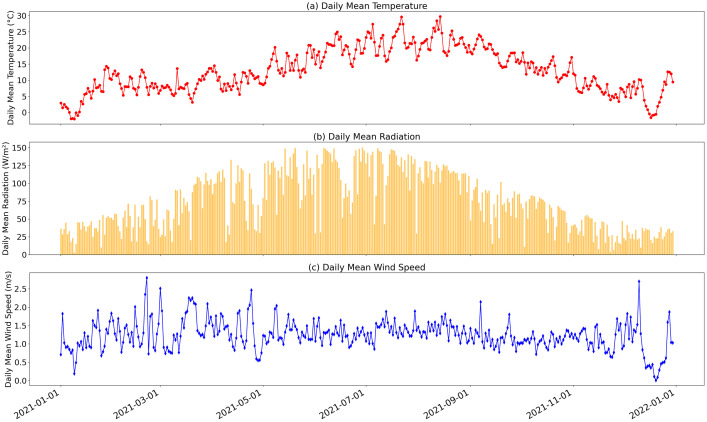
Evolution of (a) the daily mean temperature, (b) the daily mean radiation and (c) the daily mean wind speed.

The list of parameters included in each file, with their units, precision and range is shown in the following tables:

As examples of the content of the datasets, Fig. 3 shows the evolution of three of the meteorological variables along the year 2021: the daily mean temperature, the daily mean radiation and the daily mean wind speed.

## 4 Experimental Design, Materials and Methods

The original data are continuously collected by the Confederación Hidrográfica del Ebro (CHE) and published in real time through the web page of the Ebro Automatic Water Quality Information System (SAICA) [5] and the Ebro Automatic Hydrographic Information System (SAIH) [6]. Then, the CHE curates the data that are finally available to the citizens under request by variable and date. In order to facilitate their availability and reuse, these data have been gathered, pre-processed and packaged in the form of datasets. Specifically, there are four types of data in these datasets: vertical profiles of physicochemical data in the reservoir (Table 1), meteorological data in the same basin (Table 2), water level and stored water volume in the reservoir (Table 3) and water flow rate of the Queiles River downstream of the reservoir (Table 4). The materials and methods followed to collect the data of these four categories are detailed below:

• *Sub-daily vertical profiles of physicochemical data:* they are collected by a multiparametric probe (aquaDam, Adasa Systems) that is hanging from a structure located in the dam. Every 6 h, starting at noon in GMT time (1:00 p.m. in winter time, 2:00 p.m. in summer), it makes a vertical profile taking the measurements at each metre of depth from the surface to approximately 573 m.a.s.l. This level is between 2 and 3 m above the bottom outlet. The aquaDam equipment is mainly composed of an automatic positioning system, a multi-parameter probe, a management automaton and a communications system with the control centre where the information is received for subsequent study.

**Table 1:** Sub-daily vertical profiles of physicochemical data and chlorophyll concentration data.

Column	Parameter	Unit/Format	Precision	Range
0	Date–Time	YYYY-MM-DD HH:MM:SS	Second	2018-01-20 18:26:00–2022-12-22 03:23:00
1	Elevation	m.a.s.l	0.01	570.19–626.84
2	Depth	m	0.01	0.00–45.06
3	Water Temperature	°C	0.1	0.0–28.9
4	pH	–	0.01	2.36–10.55
5	ORP	mV	1	–232–503
6	Conductivity	µS cm^−1^	1	0–922
7	Dissolved oxygen	mg L^−1^	0.1	0.0–20.0
8	Turbidity	NTU	0.1	0.0–3000.0
9	Chlorophyll	µg L^−1^	0.1	0.0–500.0

**Table 2:** Daily meteorological data from the weather station located in Los Fayos (Zaragoza, Spain) municipality.

Column	Parameter	Unit/Format	Precision	Range
0	Date	YYYY-MM-DD	Day	2018-01-01–2022-12-31
1	Daily mean wind speed	m s^−1^	0.01	0.00–3.82
2	Accumulated precipitation	mm	0.1	0.0–88.5
3	Daily maximum temperature	°C	0.1	–0.9–41.7
4	Daily minimum temperature	°C	0.1	–6.6–21.9
5	Daily mean temperature	°C	0.01	–3.17–32.27
6	Date-Time maximum daily temperature achieved	YYYY-MM-DD HH:MM:SS	Minute	2018-01-01 13:45:00–2022-12-30 14:30:00
7	Date-Time minimum daily temperature achieved	YYYY-MM-DD HH:MM:SS	Minute	2018-01-01 02:30:00–2022-12-30 00:15:00
8	Daily maximum radiation	W m^–2^	0.1	0–1353
9	Daily mean radiation	W m^–2^	0.01	0–422.42
10	Date-Time Maximum daily radiation achieved	YYYY-MM-DD HH:MM:SS	Minute	2018-01-01 13:45:00–2022-12-30 14:30:00

**Table 3:** Daily level and stored water volume of the 'El Val' reservoir.

Column	Parameter	Unit/Format	Precision	Range
0	Date	YYYY-MM-DD	Day	2018-01-01–2022-12-31
1	Water reservoir total volume	hm^3^	0.001	10.869–24.12
2	Water level	m.a.s.l	0.01	604.61–619.93

**Table 4:** Daily flow of the Queiles River in Los Fayos (Zaragoza, Spain) municipality.

Column	Parameter	Unit/Format	Precision	Range
0	Daily maximum water flow	m^3^ s^–1^	0.001	0.090–5.505
1	Daily minimum water flow	m^3^ s^–1^	0.01	0.080–2.834
2	Daily mean water flow	m^3^ s^–1^	0.01	0.088–1.834
3	Date-Time maximum daily flow achieved	YYYY-MM-DD HH:MM:SS	Minute	2018-01-01 00:00:00–2022-12-30 09:00:00
4	Date-Time minimum daily flow achieved	YYYY-MM-DD HH:MM:SS	Minute	2018-01-01 22:00:00–2022-12-30 09:30:00
5	Date-Time	YYYY-MM-DD HH:MM:SS	Day	2018-01-01 00:00:00–2022-12-30 00:00:00
Column	Parameter	Unit/Format	Precision	Range

The automatic positioning system consists of a motorised drum on which the self-supporting cable that positions the probe at different depths along the vertical profile of the dam is wound. It supplies power to the probe and transmits the information to the controller. The management automaton is responsible for automatically ordering the probe positioning operations, activating the probe self-cleaning system, managing the information received, calibrating the probes and recording alarms. The communications system sends the information on each of the parameters analysed to the control centre via GPRS through a modem, via any of the current operating networks with coverage in the area).

The parameters that it registers are: date and time, level of the probe (m.a.s.l.), depth of the probe below the water surface (m), temperature (°C), pH, oxidation–reduction potential (ORP) (mV), conductivity (µS cm^–1^), dissolved oxygen (mg L^−1^), turbidity (NTU) and chlorophyll concentration (µg L^−1^).

• *Daily weather data:* the closest weather station, EMA-El Val (code EM71) [7], was selected. It is located in the municipality of Los Fayos, a few meters from the dam. It has a 6 m tower with a weather vane and an anemometer on the top, and sensors to measure temperature, radiation and pressure at mid height. Next to the tower there is a pluviometer. The variables that are available from this station are: the daily minimum (AirTempMin), maximum (AirTempMax) and mean air temperature (AirTempAvg) (°C), the daily mean wind speed (Wind) (m s^−1^), the daily maximum (RadMax) and mean (RadAvg) solar irradiation (W m^−2^) and the daily cumulative precipitation (Rain) (L m^−2^).
• *Water level and stored volume:* the monitoring station of the reservoir (code E071) [8] was selected to gather these data. It stores data of daily level (m.a.s.l.) and the stored water volume (hm³) .
• *Water flow rate of the Queiles River:* the data from the Queiles River's gauge station (code A174) [9] were gathered, which is located in the municipality of Los Fayos, a few meters downstream of the dam. In this point the water level in the Queiles River channel is measured and transformed into water flow rate considering the shape of the channel, therefore the curve that relates level and flow is updated periodically as it can change either due to natural or anthropogenic processes. Finally, the variable that is available is the flow rate (m^3^ s^−1^).

## Limitations

The vertical profiles data from 2020 to 03–28 10:04 to 2020-05-08 12:33, coinciding with the closure due to the COVID-19 pandemic, are missing.

• The dataset is limited to the selected years: from 2018, when the monitoring began, to 2022. The data continue being created and stored at the SAIH and the SAICA and anyone who follows the process described in this article can create its own updated dataset.
• The data has not been cleaned to remove or subsitute outliers, this is at the users' choice and discretion. In general they can easily detected by visual inspection, although users can implement other methods.

## Ethics Statement

The authors have read and follow the ethical requirements for publication in Data in Brief and confirm that the current work does not involve human subjects, animal experiments, or any data collected from social media platforms.

## CRediT authorship contribution statement

**María Castrillo:** Conceptualization, Data curation, Writing – original draft. **Fernando Aguilar:** Data curation, Writing – original draft. **Daniel García-Díaz:** Data curation, Writing – original draft.

## Data Availability

Dataset on sub-daily vertical profiles of physicochemical parameters and chlorophyll concentration in El Val reservoir, together with its daily meteorological data, storage state and downstream flow ( (Original data) (EDI Data Portal).

## References

[1] Castrillo M., Aguilar F., García-Díaz D. (2024). A data-driven approach for the assessment of the thermal stratification of reservoirs based on readily available data. Ecol. Inform..

[2] Chorus I., Welker M. (2021).

[3] United Nations, Department of Economic and Social Affairs (DESA), The sustainable development goals report 2022 (2022). URL https://unstats.un.org/sdgs/report/2022/The-Sustainable-Development-Goals-Report-2022.pdf.

[4] Chorus I., Welker M.. (2021). Toxic Cyanobacteria in Water.

[5] Anon. Web page from the Ebro automatic water quality information system. https://saica.chebro.es/ Accessed on March 2023.

[6] Anon. Web page from the Ebro automatic hydrographic information. Systemhttp://www.saihebro.com/ Accesed on March 2023.

[7] Anon. Web page from the Ebro automatic hydrographic information. Station EM71. http://www.saihebro.com/saihebro/index.php?url=/datos/ficha/estacion:EM71 Accesed on March 2023.

[8] Anon. Web page from the Ebro automatic hydrographic information. Station E071 http://www.saihebro.com/saihebro/index.php?url=/datos/ficha/estacion:E071 Accesed on March 2023.

[9] Anon. Web page from the Ebro automatic hydrographic information. Station A174. http://www.saihebro.com/saihebro/index.php?url=/datos/ficha/estacion:A174 Accessed on March 2023.

